# Automated chest-radiography as a triage for Xpert testing in resource-constrained settings: a prospective study of diagnostic accuracy and costs

**DOI:** 10.1038/srep12215

**Published:** 2015-07-27

**Authors:** R. H. H. M. Philipsen, C. I. Sánchez, P. Maduskar, J. Melendez, L. Peters-Bax, J. G. Peter, R. Dawson, G. Theron, K. Dheda, B. van Ginneken

**Affiliations:** 1 Diagnostic Image Analysis Group, Radboud university medical center, Department of Radiology and Nuclear Medicine Internal postal code 766, 6500 HB Nijmegen, The Netherlands; 2 Department of Radiology and Nuclear Medicine, Radboud university medical center, Department of Radiology, Internal postal code 766 6500 HB Nijmegen, The Netherlands; 3 Lung Infection and Immunity Unit and Centre for Tuberculosis Research Innovation, Division of Pulmonology, Department of Medicine, University of Cape Town Lung Institute Cape Town, South Africa

## Abstract

Molecular tests hold great potential for tuberculosis (TB) diagnosis, but are costly, time consuming, and HIV-infected patients are often sputum scarce. Therefore, alternative approaches are needed. We evaluated automated digital chest radiography (ACR) as a rapid and cheap pre-screen test prior to Xpert MTB/RIF (Xpert). 388 suspected TB subjects underwent chest radiography, Xpert and sputum culture testing. Radiographs were analysed by computer software (CAD4TB) and specialist readers, and abnormality scores were allocated. A triage algorithm was simulated in which subjects with a score above a threshold underwent Xpert. We computed sensitivity, specificity, cost per screened subject (CSS), cost per notified TB case (CNTBC) and throughput for different diagnostic thresholds. 18.3% of subjects had culture positive TB. For Xpert alone, sensitivity was 78.9%, specificity 98.1%, CSS $13.09 and CNTBC $90.70. In a pre-screening setting where 40% of subjects would undergo Xpert, CSS decreased to $6.72 and CNTBC to $54.34, with eight TB cases missed and throughput increased from 45 to 113 patients/day. Specialists, on average, read 57% of radiographs as abnormal, reducing CSS ($8.95) and CNTBC ($64.84). ACR pre-screening could substantially reduce costs, and increase daily throughput with few TB cases missed. These data inform public health policy in resource-constrained settings.

The Xpert® MTB/RIF (Xpert) test (GeneXpert, Cepheid, Sunnyvale, CA, USA) is a promising molecular test with great potential for the diagnosis of tuberculosis (TB) with high sensitivity and high specificity^1^. Since its endorsement by the World Health Organization (WHO) in December 2010, over four million tests have been procured and the yearly rate of purchased cartridges is increasing^2^. The costs for Xpert have significantly decreased with the help of external funding sources, but remain high ($13–$38)^3^,^4^,^5^,^6^,^7^,^8^,^9^,^10^,^11^, which is a hurdle for resource-constrained settings^12^. Furthermore, the current scaling up of TB diagnostics has created critical funding gaps according to the WHO Global Tuberculosis Report 2013^13^. A second disadvantage of the Xpert test is the requirement for a sputum sample. In HIV-endemic regions, this is problematic as many HIV-infected patients are unable to produce sputum^14^,^15^,^16^. Obtaining sputum from high numbers of subjects is time consuming and logistically challenging, and the majority of TB prevalence surveys to date pre-screen participants with symptoms and chest radiography (WHO Strategy 3)^13^,^17^. Thirdly, the Xpert test involves two hours of processing time, restricting daily throughput. Thus, high cost and long processing intervals limit the value and widespread uptake of Xpert as a point-of-care test. Other widely used tools for the diagnosis of active TB, which are older but reliant, are sputum smear microscopy and culture. The former is relatively cheap ($2.10 - $4.60)^6^,^9^,^11^ but has limited performance^18^. The latter is rather expensive ($5 - $15)^6^,^9^ and has a processing time of multiple weeks. Thus, both tests are suboptimal for the detection of TB in point-of-care settings. Alternative algorithms or tests are urgently needed.

Pre-screening tests have the potential to improve throughput if they are quicker to perform than Xpert, and should also reduce cost^19^. In this study, we investigated a novel pre-screening test, namely automated chest radiography (ACR), which consists of digital chest radiography assisted by automated interpretation by computer software. This test is rapid: it requires only correct positioning of the patient to acquire a radiographic image. The image is subsequently processed within 1 minute by a standard computer software program. The advantages of automatically analysing radiographs with computer software are the need of only a radiographer, and no on-site clinical officer. The latter might not always be available and is expensive. ACR is therefore not labour intensive and is faster and potentially cheaper than conventional CXR reading. A recent study by Maduskar *et al*.^20^ showed that sensitivity and specificity of computerized reading was not significantly different from reading by clinical officers.

In this study, we evaluate ACR as a pre-screening tool prior to molecular (Xpert) testing. We propose a diagnostic algorithm that is tested on data from a passive case-finding study, but could also be employed in other settings. The performance of this diagnostic algorithm is reported in terms of sensitivity, specificity, throughput and costs, and is compared with a scenario where Xpert is used for all patients and an alternate scenario in which the radiographs are read by humans.

## Methods

### Data

For this study, data from the TB-NEAT collaborative study was used^21^. All patients enrolled in this study were self-referred TB suspects presenting at a busy urban clinic in Cape Town, South Africa. All subjects provided sputum, underwent digital chest radiography on an Odelca DR unit (Delft Imaging Systems, Veenendaal, The Netherlands) and were offered voluntary testing and counselling for HIV. In the parent study, conventional radiography was used and liquid culture, and Xpert testing was performed by a trained laboratory technician in a centralized reference laboratory. Full study details have been reported elsewhere^21^. The study was carried out according to the protocol approved by the University of Cape Town Faculty of Health Sciences Research Ethics Committee (#404/2010). All patients provided written informed consent for study participation. Of the 419 recruited patients, 6 patients had missing radiographs, 22 patients had no conclusive Xpert or culture results and the software was unable to ascertain a diagnosis for 3 cases. Thus, a total of 388 patients had all information available. Culture positivity for *Mycobacterium tuberculosis* served as the reference standard. All cases were scored, blinded to any clinical information, on an integer scale from 0 to 100 by two CRRS certified “B” readers^22^,^23^ and an experienced radiologist (reader 3) for the presence of abnormalities consistent with active TB, with a score >50 considered suspicious for active TB.

### Diagnostic algorithm

**Figure 1:**
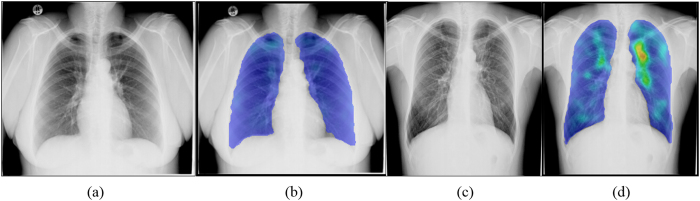
Two example CXRs with corresponding heatmaps. (**a**) Culture negative CXR with an ACR score of 18. (**b**) Corresponding abnormality heatmap. (**c**) Culture positive CXR with a subtle abnormality visible on the CXR and picked up by the CAD4TB software resulting in an ACR score of 71. (**d**) Corresponding abnormality heatmap. ACR, automated chest radiography; CXR, chest X-ray.

**Figure 2:**
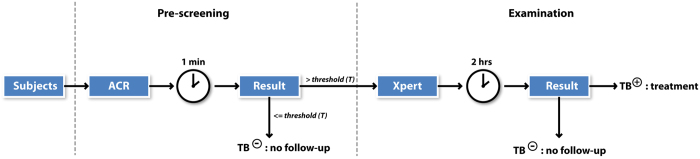
Diagnostic algorithm proposed in this paper. Subjects presenting at a TB screening unit start with a pre-screening ACR with a 1-minute computation time to determine whether follow-up Xpert test is needed. In the latter case, the Xpert test takes 2 hours to complete. ACR, automated chest radiography; TB, tuberculosis; Xpert, Xpert MTB/RIF.

All chest X-rays (CXRs) were retrospectively processed by the CAD4TB v3.07 (Diagnostic Image Analysis Group, Radboud university medical center, Nijmegen, The Netherlands) computer software. This software produces a continuous image abnormality score between 0 (normal) and 100 (highly abnormal). An example can be found in Fig. 1. Using various thresholds (*T*) on this score, we simulated the effect of using the software as a pre-screening test for molecular testing. In this simulated diagnostic algorithm, subjects with a score smaller than or equal to *T* were regarded as TB-negative, and these subjects would not undergo additional testing; otherwise, Xpert test results were used. The algorithm is schematically depicted in Fig. 2.

### Hypothetical point-of-care testing unit

For this study, we assumed a hypothetical setting where a mobile TB unit is used for passive case finding and point-of-care testing. This hypothetical unit would have one digital radiography system and three 4-cartridge GeneXpert IV machines, resulting in a capacity of 300 ACRs per day, and an Xpert testing capacity of 45 tests per day. Subjects selected for Xpert testing by the ACR pre-screening would be referred directly for Xpert testing in the same unit.

### Cost analysis

Cost analysis was limited to the point-of-care-associated costs of diagnosis. We assumed an Xpert price of $13.06, based on the FIND subsidized price for Xpert cartridges^24^. The price of ACR is determined largely by the cost of the radiography unit and the throughput. We assume a cost of $1.46 for ACR^9^,^25^. Furthermore, no increased costs were assumed for manual CXR reading. Detailed cost calculations for Xpert and ACR can be found in Table S1 and S2 of the supplementary material.

### Performance analysis

Diagnostic performance was assessed by computing sensitivity, specificity, positive predictive value (PPV) and negative predictive value (NPV) at each threshold. With the assumed costs and capacity, the cost per screened subject (CSS), the cost per notified TB case (CNTBC) as well as the throughput in cases/day was calculated. The performance of ACR was compared to CXR pre-screening with specialist readers. In this scenario, the ACR score was replaced by the composite score of specialist readings, and different thresholds (*R*) were simulated and performance was computed. We also evaluated scenarios where all HIV-infected patients would undergo Xpert testing, without receiving pre-screening. Sensitivity changes were tested for significance using the McNemar χ^2^ test (IBM SPSS Statistics 20), considering *p* < 0.05 significant.

## Results

The dataset contained 388 cases, of which 128 (33.0%) subjects were HIV-positive, 71 (18.3%) subjects were culture positive, 62 (16.0%) subjects were Xpert positive and 56 (14.4%) subjects were both culture and Xpert positive. These results are listed in Table 1. The performance, in terms of area under ROC curve, of the CAD4TB software and standalone specialist readers is comparable and not significantly different, and is shown in the supplementary Figure S1. Table 2 shows the performance of the ACR system at different thresholds. Thresholds were chosen based on the percentage selected for downstream Xpert testing shown in the second column. The first row matches the scenario where ACR is omitted and every subject receives an Xpert test. The Xpert baseline performance in terms of sensitivity and specificity was 78.9% and 98.1%, respectively, resulting in a CSS and CNTBC of $13.09, and $90.70, respectively. Significant changes in sensitivity are marked with an asterisk (*). The performance of the diagnostic algorithm combined with human reading is shown in Table 3.

### Sensitivity and specificity

The maximum achievable sensitivity with this algorithm is that of the Xpert test. With increasing ACR score thresholds, more subjects (including TB-positives) were excluded for Xpert testing, which by definition decreases sensitivity and increases specificity. The changes in sensitivity and specificity were moderate for scenarios up to *T* = 85, where 40% of subjects undergo Xpert testing: sensitivity decreased to 67.6% and specificity increased to 99.7%. This threshold gave the highest sensitivity for the maximal specificity.

### PPV and NPV

The PPV started at 90.3% and NPV at 95.4% for the Xpert test. Increasing *T* leads to less false positives and therefore increased the PPV. At the same time, the NPV decreased as there are more false negative tests. At *T* = 85, the PPV has increased to 98.0% and the NPV was 93.2%.

### Costs

The CSS and CNTBC for the Xpert test without ACR were $13.09 and $90.70, respectively. For conservative thresholds *T*, these costs increased, because the additional costs to perform ACR on the complete population outweighed the costs saved by excluding a small group for Xpert testing. The costs saved were dependent on the chosen threshold. For medium-range ACR score thresholds, CSS decreased nearly 50%: from $13.09 (*T* = 0) to $6.72 (*T* = 85), and the CNTBC decreased nearly 40% from $90.70 (*T* = 0) to $54.34 (*T* = 85). For higher thresholds, the performance decreased substantially with only a small decrease in costs.

### Throughput

Given the Xpert and ACR capacities, the daily throughput was calculated for each threshold *T*. The throughput started at 45 for the Xpert-only scenario, and increased to 150 for pre-screening with *T* = 94. The maximum capacity of the digital radiography unit was not reached due to the limited Xpert capacity.

### Performance analysis stratified by HIV status

The analysis stratified by HIV status, is shown in Tables 4 and 5. Comparing the tables, it can be seen that the performance of Xpert was significantly better in HIV-uninfected patients than in HIV-infected patients. The HIV-uninfected group showed a sensitivity of 93.9% and specificity of 99.1% for Xpert alone, and changed to 87.9% and 100%, respectively, with higher thresholds (*T* = 85). The CSS and CNTBC decreased 49% and 45%, respectively. The sensitivity and specificity of Xpert for HIV-infected subjects was 65.8% and 95.6%, respectively. For higher thresholds (*T = *85), sensitivity dropped to 50.0% and specificity increased to 98.9% (see Table 5). The CSS and CNTBC decreased by 49% and 33%, respectively.

Table 6 shows the results for a scenario where all HIV-infected and HIV-unknown subjects get Xpert testing and HIV-uninfected patients get ACR pre-screening. For thresholds up to 50% Xpert (*T = *95), the performance is better compared to a scenario where all patients get pre-screening. For example, at *T = *84 (60% Xpert), the sensitivity decreased non-significantly to 76.1% and specificity increased to 98.7%, while the CSS decreased to $8.81 (33% decrease), CNTBC to $63.30 (30% decrease) and throughput increased to 75.

### Specialist reading

The results of specialist reading are shown in Table 3. Based on *R* = 50, the specialist reader scores associated with suspicion for active TB, three readers committed 63%, 56% and 53%, respectively, of the cases for Xpert testing. The sensitivity for reader 1 was slightly higher than for reader 2 and 3: 76.1% versus 74.6%, but came with a slightly decreased specificity: 98.4% versus 99.1%. The PPV for readers 2 and 3 is better than for reader 1: 94.6% versus 91.5%. The NPV was similar for all readers, with 94.8% and 94.6%. As reader 2 and 3 sent fewer patients for Xpert testing, the CSS are slightly lower, $8.75 and $8.43 versus $9.66, respectively, as are the CNTBC: $64.04 and $61.07 versus $69.40, respectively. Comparing these numbers with the ACR threshold *T* = 60 (see Table 2), which also processed roughly 60% of the patients for Xpert, the specialist readers perform marginally better. As the radiographs were scored on a scale from 0 to 100, higher thresholds could also be used. For the scenario where roughly 30% of the subjects receive Xpert testing, the automated algorithm outperforms reader 1, shows similar results for reader 3; however it is inferior to reader 2 (see Table 2).

## Discussion

Following WHO recommendations for molecular based testing in 2010, the procurement of the GeneXpert MTB/RIF systems in resource-constrained countries is high and is still expanding. However, available funds are often limited, and the WHO has also highlighted the need for cost-saving diagnostic pathways. Our results have shown that ACR pre-screening may offer a solution: being cheaper, faster, with only a moderate decrease in sensitivity, and the benefit of increased throughput, as compared with Xpert testing. Using the ACR pre-screening algorithm at *T* = 85, only 40% of the patients would be sent for a downstream Xpert test, and the CSS and CNTBC were less than 51% and 60% of the original cost, respectively.

In this study, the benefit of ACR came at the cost of eight additionally missed TB cases. This can be attributed to the limitations of radiography screening itself in addition to the use of *automated* pre-screening. Of these eight cases, six were HIV-infected, and it has been previously reported that chest radiography is less sensitive in immunocompromised patients^26^,^27^. This effect was also seen in the results by HIV stratification in Tables 4 and 5. The performance of the diagnostic algorithm among HIV-uninfected patients is considerably better than in HIV-infected patients, and consideration should be applied for different thresholds for both groups. For example pre-screening might be considered only for HIV-uninfected patients (Table 6). This would still reduce CSS and CNTBC with more than 30% (*T = *84), with only a marginally non-significant reduction in sensitivity (two additionally missed TB cases). Comparing the automated system to specialist readers, the performance of the algorithm is slightly inferior with medium-range thresholds, but comparable for high thresholds, although the performance among readers differed. However, although not modelled in this study, specialist reading is more time consuming, requires training and also increases costs.

The proposed pre-screening diagnostic algorithm is fully automated and requires only an on-site radiographer. The availability of automated analysis software obviates the need for a radiologist or clinical officer and limits the throughput to that of the radiography unit only. In our simulations, we kept the cost of ACR and manual reading constant at $1.46, and this amount did not change with altered throughput nor did it affect the main outcomes of the study.

Besides cost-reduction, pre-screening can substantially increase throughput, although it would remain limited by the Xpert machine’s capacity. This may be particularly useful in screening settings, as sputum collection would not be needed for many potential TB subjects. Additionally, given that the average test duration is reduced with most patients receiving a negative radiology test result within a few minutes, this would obviate a 2-hour wait for Xpert results.

We predict that, in a typical screening setting, pre-screening with ACR could increase patient throughput by up to 250%. Another advantage of *automated* reading is that different thresholds with objective and reproducible results could be chosen. Thus, according to the setting used, a threshold could be chosen to obtain the desired sensitivity, specificity or throughput. This makes ACR highly valuable in resource-constrained settings, and this proposed diagnostic pathway can save both cost and time in a point-of-care setting. We hypothesize that the algorithm’s value is even higher in active case-finding scenarios and sputum-scarce cohorts, but future studies are needed.

Compared to a recent study by Muyoyeta *et al*.^28^, which uses a previous version of the CAD4TB software on presumptive TB patients with Xpert as reference, the current software showed improved performance and supported their findings that ACR has a potential role in TB screening. Additionally, the current software showed better performance values than reported in a previous study by Maduskar *et al*.^20^.

The proposed diagnostic algorithm does not account for patients with high ACR scores but negative Xpert results: it is likely that these patients either have a false negative Xpert test or may have disease other than TB. Furthermore, current costs analysis was limited to the costs of diagnosis only and does not take into account the cost of treatment and misdiagnosed patients. This group of patients should be flagged to receive further attention based on their ACR scores. In general, basic automated screening for abnormalities could prompt referral mechanisms for higher levels of care.

In conclusion, ACR pre-screening before sputum Xpert molecular testing is a promising new advance in TB diagnostics. The ACR algorithm can significantly reduce cost and substantially increase throughput, while maintaining high sensitivity. This makes it a potentially highly valuable diagnostic tool in resource-constrained countries.

## Additional Information

**How to cite this article**: Philipsen, R.H.H.M. *et al*. Automated chest-radiography as a triage for Xpert testing in resource-constrained settings: a prospective study of diagnostic accuracy and costs. *Sci. Rep*. **5**, 12215; doi: 10.1038/srep12215 (2015).

## Supplementary Material

Supplementary Information

## Figures and Tables

**Table 1:** Study data demographics and statistics.

	*N* (%)
Total	388
Gender	
Male	235 (60.6)
Female	153 (39.4)
Mean age (SD)	39.9 (12.0)
HIV status	
positive	128 (33.0)
negative	252 (64.9)
unknown	8 (2.1)
Bacteriological findings	
Culture positive	71 (18.3)
Xpert positive	62 (16.0)
Culture positive and Xpert positive	56 (14.4)

SD, standard deviation.

**Table 2:** Performance results of the proposed diagnostic algorithm with different ACR score thresholds, *T*.

ACR threshold *T*	Selected for Xpert (%)	Sensitivity (%)	Specificity (%)	PPV (%)	NPV (%)	CSS ($)	CNTBC ($)	Throughput per day
0	100	78.9	98.1	90.3	95.4	13.09	90.70	45
22	90	76.1	98.4	91.5	94.8	13.23	95.09	50
36	80	76.1	99.1	94.7	94.8	11.91	85.64	56
47	70	73.2	99.1	94.5	94.3	10.63	79.36	64
60	60	70.4*	99.1	94.3	93.7	9.32	72.33	75
73	50	70.4*	99.1	94.3	93.7	7.97	61.86	90
85	40	67.6*	99.7	98.0	93.2	6.72	54.34	113
94	30	63.4*	99.7	97.8	92.4	5.34	46.04	150
97	20	49.3*	99.7	97.2	89.8	4.09	45.36	225

The first row matches a scenario where all subjects undergo Xpert and no ACR. Throughputs are based on an Xpert capacity of 45/day and an ACR capacity of 300/day. Costs for Xpert and ACR are $13.09 and $1.46 respectively.

ACR, automated chest radiography; CNTBC, cost per notified TB case; CSS, cost per screened subject; NPV, negative predictive value; PPV, positive predictive value; Xpert, Xpert MTB/RIF.

^*^Sensitivity significantly different from Xpert standalone, *T* = 0. (*p* < 0.05 considered significant).

**Table 3:** Performance results of the proposed diagnostic algorithm with human reading instead of ACR.

	Reader threshold *R*	Selected for Xpert (%)	Sensitivity (%)	Specificity (%)	PPV (%)	NPV (%)	CSS ($)	CNTBC ($)	Throughput per day
Reader 1	50	63	76.1	98.4	91.5	94.8	9.66	69.40	71
Reader 1	64	32	57.7	99.1	93.2	91.3	5.68	53.73	140
Reader 2	50	56	74.6	99.1	94.6	94.6	8.75	64.04	80
Reader 2	69	31	69.0	99.4	96.1	93.5	5.47	53.15	147
Reader 3	50	53	74.6	99.1	94.6	94.6	8.43	61.07	85
Reader 3	70	28	63.4	99.4	95.7	92.4	5.14	44.30	160

Throughputs are based on an Xpert capacity of 45/day. For each reader, two thresholds, *R*, are shown: normal/abnormal CXR (*R = *50) and a more progressive threshold (~30% Xpert).

ACR, automated chest radiography; CNTBC, cost per notified TB case; CSS, cost per screened subject; CXR, chest X-ray; NPV, negative predictive value; PPV, positive predictive value; Xpert, Xpert MTB/RIF.

**Table 4:** Performance results of the proposed diagnostic algorithm with different ACR score thresholds, *T*, for HIV-uninfected (*N* = 252) patients only.

ACR threshold *T*	Selected for Xpert (%)	Sensitivity (%)	Specificity (%)	PPV (%)	NPV (%)	CSS ($)	CNTBC ($)
0	100	93.9	99.1	93.9	99.1	13.09	106.41
24	90	93.9	99.1	93.9	99.1	13.25	107.72
42	80	93.9	99.5	96.9	99.1	11.95	97.16
50	70	93.9	99.5	96.9	99.1	10.60	86.19
67	60	90.9	99.5	96.8	98.6	9.30	78.15
76	50	90.9	99.5	96.8	98.6	8.01	67.24
85	40	87.9	100.0	100.0	98.2	6.71	58.28
93	30	78.8*	100.0	100.0	96.9	5.41	52.41
96	20	69.7*	100.0	100.0	95.6	4.11	45.02

The first row matches a scenario where all subjects undergo Xpert and no ACR. ACR, automated chest radiography; CNTBC, cost per notified TB case; CSS, cost per screened subject; NPV, negative predictive value; PPV, positive predictive value; Xpert, Xpert MTB/RIF.

^*^Sensitivity significantly different from Xpert standalone, *T* = 0. (*p* < 0.05 considered significant).

**Table 5:** Performance results of the proposed diagnostic algorithm with different ACR score thresholds, *T*, for HIV-infected (*N* = 128) patients only.

ACR threshold *T*	Selected for Xpert (%)	Sensitivity (%)	Specificity (%)	PPV (%)	NPV (%)	CSS ($)	CNTBC ($)
0	100	65.8	95.6	86.2	86.9	13.09	67.02
17	90	60.5	96.7	88.5	85.3	13.22	73.58
35	80	60.5	97.8	92.0	85.4	11.89	66.18
44	70	55.3	97.8	91.3	83.8	10.66	65.00
54	60	52.6*	97.8	90.9	83.0	9.33	59.74
73	50	52.6*	97.8	90.9	83.0	8.01	51.23
85	40	50.0*	98.9	95.0	82.4	6.68	44.97
95	30	44.7*	98.9	94.4	80.9	5.35	40.25
98	20	34.2*	100.0	100.0	78.3	4.12	40.56

The first row matches a scenario where all subjects undergo Xpert and no ACR.

ACR, automated chest radiography; CNTBC, cost per notified TB case; CSS, cost per screened subject; NPV, negative predictive value; PPV, positive predictive value; Xpert, Xpert MTB/RIF.

^*^Sensitivity significantly different from Xpert standalone, *T* = 0. (*p* < 0.05 considered significant).

**Table 6:** Performance results of the proposed diagnostic algorithm with different ACR score thresholds, *T*, where only HIV-uninfected and HIV-unknown subjects get ACR pre-screening and all HIV-infected subjects get Xpert testing.

ACR threshold *T*	Selected for Xpert (%)	Sensitivity (%)	Specificity (%)	PPV (%)	NPV (%)	CSS ($)	CNTBC ($)	Throughput per day
0	100	78.9	98.1	90.3	95.4	13.09	90.70	45
30	90	78.9	98.4	91.8	95.4	12.72	88.15	50
49	80	78.9	98.4	91.8	95.4	11.41	79.04	56
70	70	77.5	98.4	91.7	95.1	10.13	71.43	64
84	60	76.1	98.7	93.1	94.8	8.81	63.30	75
95	50	70.4*	98.7	92.6	93.7	7.49	58.15	90
99	42	59.2*	98.7	91.3	91.5	6.45	59.57	107
100	33	35.2*	98.7	86.2	87.2	5.27	81.75	136

The first row matches a scenario where all subjects undergo Xpert and no ACR.

ACR, automated chest radiography; CNTBC, cost per notified TB case; CSS, cost per screened subject; NPV, negative predictive value; PPV, positive predictive value; Xpert, Xpert MTB/RIF.

^*^Sensitivity significantly different from Xpert standalone, *T* = 0. (*p* < 0.05 considered significant).
