# Supplementary material for: Automated chest-radiography as a triage for Xpert testing in resource-constrained settings: a prospective study of diagnostic accuracy and costs
Source: Sci Rep. 2015 Jul 27;5:12215. doi: 10.1038/srep12215 (PMC4515744; doi:10.1038/srep12215)
Supplement: Supplementary Information [file srep12215-s1.pdf]

Automated chest-radiography as a triage for Xpert testing in resource-constrained settings: a prospective study of diagnostic accuracy and costs

R.H.H.M. Philipsen, C.I. Sánchez, P. Maduskar, J. Melendez, L. Peters-Bax, J.G. Peter, R. Dawson, G. Theron, K. Dheda, and B. van Ginneken

Table S1 shows the cost estimates for the GeneXpert MTB/RIF (Xpert) test. A depreciation period of 5 years is assumed and the average costs per cartridge are calculated based on a usage of 5 years. The cost for one Xpert test is $13·06. The costs for automated chest radiography (ACR) are summarized in Table S2. A depreciation period of 10 years is assumed and the average cost for one ACR is calculated to be $1·46.

The performance of the CAD4TB v3.07 software, human readers and Xpert test, in terms of area under the receiver operator characteristic curve (A_z), using sputum culture reference is shown in Fig S1. The software has A_z = 0.79 and the human readers have A_z = 0.76, A_z = 0.81 and A_z = 0.77, respectively. The sensitivity/specificity of the Xpert test is plotted for reference. Pairwise comparison with bootstrap estimation of the performance of the human readers with the computer software was performed, considering p<0.05 significant. The performance of all human readers compared to CAD4TB was not significantly different (p=0.91, p=0.11, p=0.70, respectively).

Table S1. Cost estimates GeneXpert MTB/RIF[1]

Row	Category	Item	Cost, number of days, tests or cartridges	Source
A	Equipment	GeneXpert IV module	US$ 17 500·00	Ref [S1]
B		Shipping	US$ 1 000·00	Ref [S1]
C		Power supply unit	US$ 1 200·00	Ref [S1]
D		Printer	US$ 200·00	Ref [S1]
E	Maintenance	Calibration kit after 1st year	US$ 450·00	Ref [S1]
F		Annual warranty after 2nd year	US$ 2 900·00	Ref [S1]
G	Consumables	Cost per cartridge	US$ 9·98	Ref [S1]
H		Shipment per cartridge	US$ 1·20	Ref [S1]
I	Productivity	Number of working days per year	250	Ref [S1,S3]
J		Average number of test per instrument per day (year 1)	8	Ref [S1,S3]
K		Average number of test per instrument per day (year 2 and beyond)	15	Ref [S1,S3]
L		Number of cartridges (year 1)	2 000	I*J
M		Number of cartridges (year 2 and beyond)	3 750	I * K
N	Human resources	Annual salary for technician*	US$ 5 500·00	Ref [S2,S3]
O		Training and monitoring	US$ 1 000·00	Assumed
P	Investment costs: year 1		US$ 19 900·00	A + B + C + D
Q	Running costs: year 1		US$ 22 810·00	E + (G+H)*L + N + O
R	Running costs: year 2		US$ 44 825·00	F + (G+H)*M + N + O
S	Depreciation period in years		5	Assumed
T	Average cartridge cost first 5 years		US$ 13·06	(P + Q + (S-1)*R)/(L+(S-1)*M)

* Based on 2 hours work needed per day

Table S2. Cost estimates automated chest radiography (ACR)

Row	Category	Item	Cost, number of days or tests	Source
A	Equipment	Direct digital X-ray	US$ 232 452·50	Ref [S2]
B		PACS	US$ 10 626·40	Ref [S2]
C		Shipment	US$ 3 320·75	Ref [S2]
D		CAD4TB software	US$ 13 283·00	Ref [S2]
E		Training & TA	US$ 3 320·75	Ref [S2]
F	Running costs	Annual maintenance cost	US$ 11 290·55	Ref [S2]
G		Annual maintenance and upgrade CAD4TB	US$ 2 656·60	Ref [S2]
H		Computer memory	US$ 415·09	Ref [S2]
I		Power supply	US$ 3 984·90	Ref [S2]
J	Productivity	Number of working days per year	250	Ref [S1-S3]
K		Average number of ACR per day	200	Assumed
L		Number of ACR per year	50 000	J*K
M	Human resources	Annual salary for technician	US$ 27 500·00	Ref [S2,S3]
N		Training and monitoring	US$ 1 000·00	Assumed
O	Investment costs		US$ 263 003·40	A + B + C + D + E
P	Yearly running costs		US$ 46 847·14	F + G + H + I + M + N
Q	Depreciation period in years		10	Ref [S2]
R	Average ACR cost first 10 years		US$ 1·46	(O/Q + P) / L

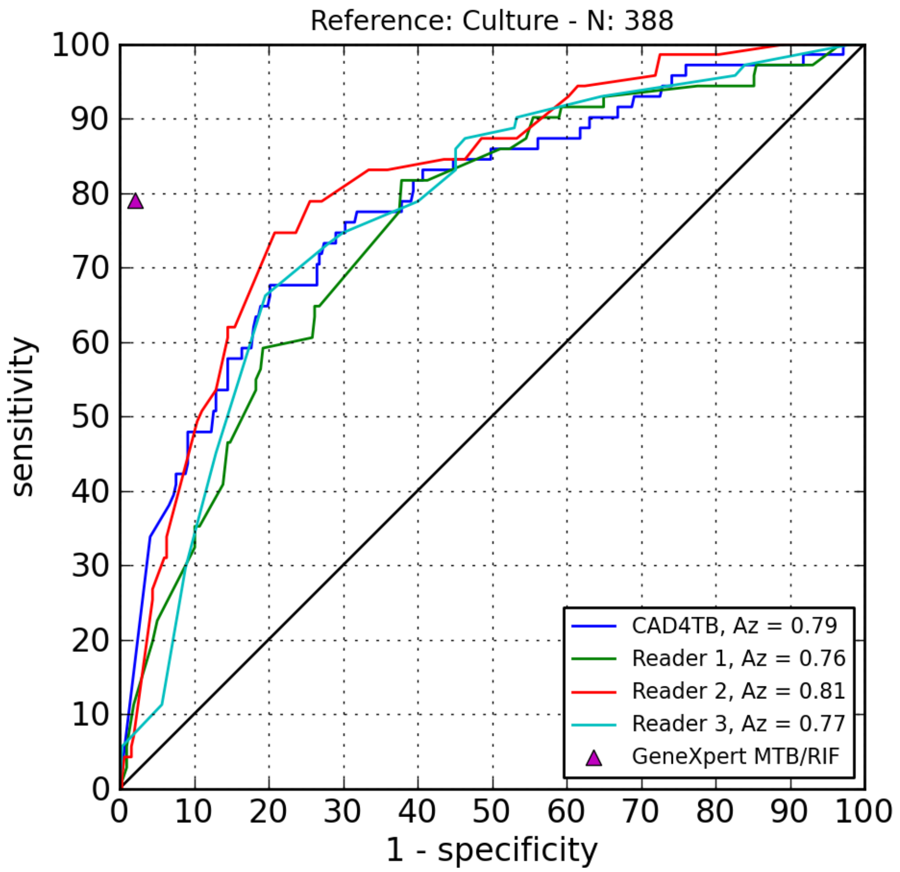

Figure S1. Receiver operating characteristic curves for CAD4TB and human readers. The purple triangle shows the performance of the Xpert test. Culture results are used as reference.

References

S1 World Health Organization. Xpert MTB/RIF implementation manual. Available at: http://apps.who.int/-iris/bitstream/10665/112469/1/9789241506700_eng.pdf?ua=1, Accessed June 2014.

S2 CheckTB! Economics of TB Screening Simulation. Available at: http://checktb.com/-index.php?option=com_wrapper&view=wrapper&Itemid=171&lang=en.

S3 Theron G, Pooran A, Peter J, van Zyl-Smit R, Mishra HK, Meldau R, et al. Do adjunct TB tests, when combined with Xpert MTB/RIF, improve accuracy and the cost of diagnosis in a resource-poor setting? 2011;40:161–168.
